# Combined BTX-A and Collagen Membrane in Benign Parotid Enucleation: A Comparative Cohort Study

**DOI:** 10.3390/cmtr19020023

**Published:** 2026-04-24

**Authors:** Giuseppe Consorti, Enrico Betti, Mariagrazia Paglianiti, Lisa Catarzi, Gabriele Monarchi, Massimiliano Gilli, Stefania Troise, Carlos Miguel Chiesa-Estomba, Luigi Angelo Vaira, Giulio Cirignaco

**Affiliations:** 1 Division of Maxillofacial Surgery, Department of Neurological Sciences, Marche University Hospitals-Umberto I, Via Conca 71, 60126 Ancona, Italy; giuseppe.consorti@ospedaliriuniti.marche.it (G.C.); dr.enricobetti@gmail.com (E.B.); mgpaglianiti@gmail.com (M.P.); 2 Department of Clinical Specialistic and Dental Sciences, Marche Polytechnic University, 60126 Ancona, Italy; 3 Section of Maxillo-Facial Surgery, Department of Medicine, University of Siena, Viale Bracci, 53100 Siena, Italy; lisa.catarzi@gmail.com; 4 Maxillo-Facial Surgery Unit, Santa Maria Della Misericordia Hospital, San Sisto, 06129 Perugia, Italy; gabriele.monarchi@gmail.com (G.M.); massimilianogilli0@gmail.com (M.G.); 5 Maxillofacial Surgery Unit, Department of Neurosciences, Reproductive and Odontostomatological Sciences, University of Naples Federico II, 80138 Naples, Italy; stefy.troise@gmail.com; 6 Department of Otorhinolaryngology-Head & Neck Surgery, Hospital Universitario Donostia, 20001 San Sebastian, Spain; chiesaestomba86@gmail.com; 7 Maxillofacial Surgery Operative Unit, Department of Medicine, Surgery and Pharmacy, University of Sassari, Viale San Pietro 43/B, 07100 Sassari, Italy; lavaira@uniss.it

**Keywords:** parotid gland neoplasms, extracapsular dissection, botulinum toxin type A, sialocele, Frey syndrome

## Abstract

Benign parotid tumors are increasingly treated with parenchyma-sparing extracapsular enucleation, yet postoperative salivary collections and Frey syndrome can still generate clinically relevant morbidity; we evaluated whether a standardized intraoperative bundle combining intraparotid botulinum toxin A (BTX-A) and bovine collagen membrane interposition is associated with fewer complications than standard enucleation alone. In this retrospective comparative cohort at a tertiary Head and Neck Surgery Unit, consecutive adults undergoing extracapsular enucleation for pleomorphic adenoma or Warthin tumor (2010–2025) were allocated by institutional era-based protocol to Group A (2010–2017, standard enucleation) or Group B (2018–2025, enucleation plus intraoperative intraparotid BTX-A 50 IU and bovine collagen membrane placement over the repaired parotid fascia). Prespecified endpoints were sialocele/salivary fistula, surgical-site infection (SSI) within 30 days, and clinically recorded Frey syndrome within 6 months; effect sizes with 95% confidence intervals were reported. A total of 188 patients were analyzed (94 per group). Sialocele occurred in 20/94 (21.3%) in Group A versus 2/94 (2.1%) in Group B [Relative Risk (RR) 0.10]. SSI occurred in 14/94 (14.9%) versus 2/94 (2.1%) (RR 0.143), and clinically recorded Frey syndrome in 18/94 (19.1%) versus 4/94 (4.3%) (RR 0.222). This combined protocol was associated with lower complication rates through 6 months; prospective controlled studies with standardized Frey assessment and longer follow-up are warranted.

## 1. Introduction

Benign tumors are the most common parotid neoplasms with pleomorphic adenoma and Warthin tumor representing the most frequent histologic subtypes usually encountered [1]. Surgical excision remains the definitive treatment; however, the optimal extent of parotid resection has progressively evolved. In appropriately selected benign lesions, contemporary surgery increasingly favors conservative, nerve-sparing techniques—such as extracapsular dissection and enucleation—aimed at limiting facial nerve manipulation, reducing operative morbidity, and preserving functioning glandular parenchyma while maintaining satisfactory oncologic safety for benign disease [2,3]. This transition has been supported by evidence that less extensive resections can achieve favorable functional outcomes with acceptable recurrence profiles when careful patient selection and meticulous technique are applied [4,5,6,7].

Despite these advantages, conservative parotid surgery is associated with a characteristic pattern of postoperative morbidity that is clinically relevant and often underestimated. By preserving secretory tissue and leaving a functioning residual parotid remnant, enucleation may predispose to early saliva-related wound complications, particularly sialocele and salivary fistula [8]. Even when absolute incidence is modest in some series, these events are clinically meaningful because they frequently require repeated aspirations and compressive dressings, increase unplanned outpatient visits, and delay recovery. In parallel, Frey syndrome is not only a cosmetic complaint but a functional, patient-reported sequela that may persist and impair quality of life. Therefore, prevention strategies that reduce the management burden of salivary collections and mitigate late gustatory symptoms are clinically relevant in contemporary conservative parotid surgery. These events can prolong drainage, necessitate repeated aspirations and compressive dressings, increase outpatient visits, and delay recovery [9,10,11,12]. Beyond the direct burden of management, persistent salivary collections may compromise wound stability and create a permissive environment for bacterial proliferation, potentially contributing to surgical-site infection (SSI) and additional antibiotic exposure [13]. In parallel, Frey syndrome remains among the most troublesome late sequelae of parotid surgery. Caused by aberrant regeneration of parasympathetic fibers toward cutaneous sweat glands, it manifests as gustatory sweating and flushing over the parotid region and can meaningfully affect quality of life even when facial nerve function is preserved [12,14,15]. Reported rates of salivary complications and Frey syndrome vary widely across studies due to differences in operative extent, use of interposition techniques, definitions, and ascertainment methods; accordingly, clinically relevant symptom-based endpoints remain important for real-world care.

Preventive strategies have traditionally targeted one of two mechanisms: temporary reduction in salivary output during the vulnerable early healing phase or mechanical separation between the parotid bed and the overlying skin to reduce aberrant reinnervation. Botulinum toxin A (BTX-A) decreases salivary secretion by inhibiting acetylcholine release at cholinergic neuroglandular junctions and has an established therapeutic role in the management of postoperative sialocele and salivary fistula. Building on this rationale, prophylactic intraparotid BTX-A delivered intraoperatively has been explored [16,17,18] to mitigate saliva-related complications after parotid resections, with reports suggesting reduced sialocele rates and postoperative drainage without an apparent increase in facial nerve morbidity.

Conversely, Frey syndrome prevention has been approached through interposition techniques designed to physically dissociate regenerating parasympathetic fibers from dermal sweat glands, superficial musculoaponeurotic system (SMAS)-based reconstruction, fat grafting, and biologic materials—including acellular dermal matrices and collagen-based membranes—have been applied to the parotid bed with the aim of reducing both subjective symptoms and objective Minor test positivity. However, heterogeneity in materials, fixation methods, and diagnostic criteria has limited consensus, and no single approach has been universally adopted as a reproducible standard in everyday practice [19,20,21,22].

Notably, most strategies are deployed as stand-alone measures and therefore address either early salivary morbidity or later gustatory sweating rather than both. To our knowledge, the combined intraoperative use of prophylactic intraparotid BTX-A and bovine collagen membrane interposition as a standardized adjunctive bundle after benign parotid tumor enucleation has not been specifically evaluated.

Within this framework, a dual-mechanism intraoperative strategy is conceptually attractive. We hypothesized that intraparotid BTX-A infiltration performed at the end of surgery could suppress salivary output during the critical early phase of wound healing, while a bovine collagen membrane placed over the repaired parotid fascia could provide durable separation of tissue planes by recreating a stable fascial-like interposition layer over the parotid bed, thereby improving wound interface stability and reducing the likelihood of aberrant parasympathetic reinnervation.

The primary objective of this study was to evaluate whether a standardized intraoperative protocol reduces clinically meaningful postoperative morbidity after benign parotid tumor enucleation, focusing on saliva-related wound complications (sialocele/salivary fistula) and clinically recorded Frey syndrome as endpoints that drive outpatient burden and patient-reported quality of life.

## 2. Materials and Methods

The study was conducted in accordance with the ethical standards as laid down in the 1964 Declaration of Helsinki and its later amendments or comparable ethical standards. To ensure patient confidentiality, all personal identifiers were removed or coded during data collection, and access to sensitive information was restricted to authorized personnel only.

Consecutive adult patients (≥18 years) who underwent conservative removal of a benign parotid tumor by extracapsular enucleation between January 2010 and December 2025 were screened. To ensure a homogeneous cohort, eligibility was restricted to primary benign epithelial tumors with definitive histopathologic diagnosis of pleomorphic adenoma or Warthin tumor on final pathology.

Exclusion criteria were predefined to minimize confounding related to wound healing and salivary morbidity and included diabetes mellitus, malignant disease on final histology, recurrent tumors, previous parotid surgery, prior sialadenitis, and any history of postoperative sialocele or salivary fistula. Patients were also excluded if follow-up was insufficient to assess the prespecified endpoints through 6 months.

For Warthin tumor, surgery was performed according to institutional practice in selected cases (e.g., symptomatic lesions, documented growth, diagnostic uncertainty, or patient preference), while asymptomatic stable lesions were commonly observed.

Patients were allocated into two cohorts according to the standardized intraoperative protocol adopted during the study period. Group A comprised patients treated from 2010 to 2017 with standard extracapsular enucleation alone. Group B comprised patients treated from 2018 to 2025 with extracapsular enucleation followed by intraoperative intraparotid botulinum toxin A (BTX-A) infiltration and placement of a bovine collagen membrane over the repaired parotid fascia.

All procedures were carried out under general anesthesia using a standard parotid approach [23]. A modified Blair incision was used with subplatysmal flap elevation to expose the parotid fascia. After identifying the tumor and planning safe margins, extracapsular enucleation was performed by developing a dissection plane immediately outside the tumor capsule using a combination of blunt and sharp dissection under magnification, with the goal of preserving uninvolved parotid parenchyma. Facial nerve branches encountered within the operative field were identified and preserved; the main trunk was dissected only when required by tumor location and exposure. Meticulous hemostasis was achieved (primarily bipolar cautery). At the completion of tumor removal, the parotid fascia was closed with interrupted absorbable sutures to reduce dead space prior to drain placement. In Group B, once tumor removal and hemostasis were completed, onabotulinumtoxinA (BOTOX^®^; Allergan, an AbbVie Company, Irvine, CA, USA) diluted in sterile saline was injected into the residual parotid parenchyma at 4–5 evenly distributed intraglandular sites for a total dose of 50 IU and never repeated after surgery. The injection was performed at the end of resection to maximize diffuse parenchymal exposure while avoiding superficial placement, consistent with prophylactic intraparotid approaches described for salivary complication prevention.

After BTX-A infiltration, the parotid fascia was closed with interrupted 4-0 absorbable sutures. A bovine collagen membrane (Lyoplant^®^, B. Braun, Germany) was then tailored and positioned over the repaired fascia with at least a 2 cm overlap beyond the dissection margins and secured with interrupted absorbable sutures to minimize displacement, providing stable interposition and recreating a fascial-like barrier between the parotid bed and the overlying tissues. A closed-suction drain was placed in all patients and managed according to institutional protocol. Drains were removed when the daily output was <30 mL/day. Standard postoperative instructions, wound care, and antibiotic policies were applied according to institutional practice and remained consistent across the study period. Postoperative follow-up was standardized for both groups, with scheduled visits at approximately 1, 3, and 6 months. At each visit, the operative site was examined for evidence of salivary complications, infection, and symptoms consistent with Frey syndrome. Facial nerve function was assessed in the immediate postoperative period and at follow-up visits using a branch-specific clinical examination.

The prespecified primary endpoints were saliva-related wound complications (sialocele and/or salivary fistula), surgical-site infection (SSI), and clinically recorded Frey syndrome within 6 months. A sialocele was defined as a postoperative, clinically evident fluctuant collection in the operative field consistent with salivary accumulation, confirmed by the treating surgeon and requiring aspiration and/or compressive management. A salivary fistula was defined as persistent external salivary leakage through the wound or drain site beyond the expected immediate postoperative period, documented on clinical examination and requiring continued local wound care and/or pressure dressings. When applicable, cases were classified according to the predominant clinical manifestation at presentation.

SSI was defined as an infection involving the operative site occurring within 30 days of surgery, diagnosed when purulent drainage was present and/or when a surgeon-diagnosed infection occurred in association with local inflammatory signs (erythema, warmth, tenderness, swelling) and/or systemic features requiring antibiotic therapy, with or without microbiologic confirmation from an aseptically obtained culture. This 30-day timeframe was used as the standard postoperative window for SSI reporting [23,24,25]. Frey syndrome was assessed during follow-up and recorded as clinically evident symptoms and/or objective confirmation when available. Clinically recorded Frey syndrome was defined as patient-reported gustatory sweating and/or flushing over the parotid region documented at follow-up visits. Objective Frey syndrome was defined as a positive Minor’s starch–iodine test when performed as part of routine clinical care in patients with symptoms or clinical suspicion. Minor testing was not protocolized for all patients; therefore, for analysis, Frey syndrome was considered present when clinically recorded symptoms were reported, with objective confirmation when available. Secondary safety outcomes included postoperative hematoma and facial nerve dysfunction. Facial nerve deficits were categorized as transient or permanent according to persistence at follow-up.

### Statistical Analysis

All analyses were two-sided, with statistical significance set at *p* < 0.05. Continuous variables were summarized as mean ± standard deviation and compared between groups using Student’s *t*-test. Categorical variables were reported as counts and percentages and compared using Fisher’s exact test when expected cell counts were low; chi-square testing was reserved for comparisons in which expected counts were adequate. In addition to *p*-values, absolute risk differences with 95% confidence intervals were calculated for binary endpoints to support clinical interpretation of effect magnitude. To aid clinical interpretability, numbers needed to treat (NNT) were calculated from absolute risk reductions. Finally, given the era-based allocation, E-values were calculated for relative risks to quantify the robustness of observed associations to potential unmeasured confounding. E-values were computed on the relative-risk scale following the approach of VanderWeele and Ding, and the ‘upper CI’ E-value was derived from the confidence-limit closest to the null (RR = 1).

## 3. Results

A total of 188 patients met the eligibility criteria and were included in the analysis, with 94 patients in each cohort according to the institutional protocol adopted during the study period. Group A comprised patients treated with standard extracapsular enucleation alone between 2010 and 2017, whereas Group B comprised patients treated between 2018 and 2025 with extracapsular enucleation followed by intraoperative intraparotid botulinum toxin A (BTX-A) infiltration and placement of a bovine collagen membrane over the repaired parotid fascia. Baseline clinical comparability between cohorts was supported by the absence of statistically meaningful differences in demographic or tumor characteristics. Mean age was 51.3 ± 11.2 years in Group A and 52.1 ± 10.8 years in Group B (*p* = 0.619), and sex distribution was similar (44/50 vs. 46/48 male/female; *p* = 0.884). Histopathologic composition was also comparable, with pleomorphic adenoma representing most lesions in both groups (62/94 [66%] in Group A and 58/94 [62%] in Group B) and Warthin tumor accounting for the remaining cases (32/94 [34%] and 36/94 [38%], respectively; *p* = 0.649) (Table 1).

At 6-month follow-up, the combined intraoperative protocol was associated with lower complication rates across all prespecified endpoints (Table 2).

The most pronounced difference involved saliva-related wound morbidity. Sialocele occurred in 20/94 patients (21.3%) in Group A compared with 2/94 patients (2.1%) in Group B (Fisher’s exact *p* = 0.0000478), corresponding to an absolute risk difference of −19.1% (95% CI −30.0 to −6.8) and concordant effect size estimates (relative risk 0.10 [95% CI 0.024–0.416] and odds ratio 0.080 [95% CI 0.018–0.355]). This corresponded to an estimated number needed to treat (NNT) of 5.2.

**Figure 1:**
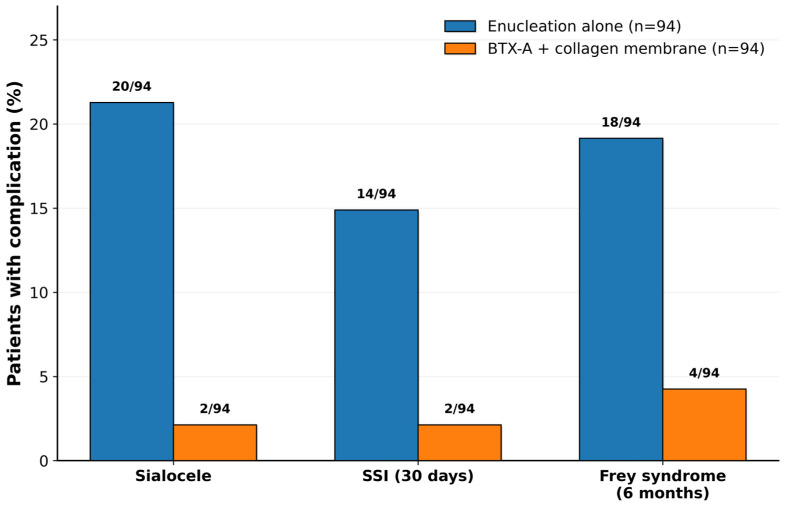
Postoperative complication rates by study cohort. Proportion of patients with sialocele, surgical-site infection (within 30 days), and Frey syndrome (within 6 months) in Group A (standard extracapsular enucleation; *n* = 94) versus Group B (extracapsular enucleation plus intraoperative intraparotid botulinum toxin A infiltration and bovine collagen membrane placement; *n* = 94). Bars represent percentages calculated from observed event counts (sialocele: 20/94 vs. 2/94; SSI: 14/94 vs. 2/94; Frey syndrome: 18/94 vs. 4/94).

Surgical-site infection within 30 days was recorded in 14/94 patients (14.9%) after standard enucleation and in 2/94 patients (2.1%) after the combined protocol (Fisher’s exact *p* = 0.00283), with an absolute risk difference of −12.8% (95% CI −22.9 to −1.7), a relative risk of 0.143 (95% CI 0.033–0.611), and an odds ratio of 0.124 (95% CI 0.027–0.563); the corresponding NNT estimate was approximately 8. Clinically recorded Frey syndrome at 6 months was observed in 18/94 patients (19.1%) following standard enucleation and in 4/94 patients (4.3%) after BTX-A infiltration and collagen membrane placement (Fisher’s exact *p* = 0.00247), translating into an absolute risk difference of −14.9% (95% CI −26.6 to −2.0), a relative risk of 0.222 (95% CI 0.078–0.632), and an odds ratio of 0.188 (95% CI 0.061–0.578); the corresponding NNT estimate was approximately 7. Complication rates by cohort are summarized visually in Figure 1.

**Figure 2:**
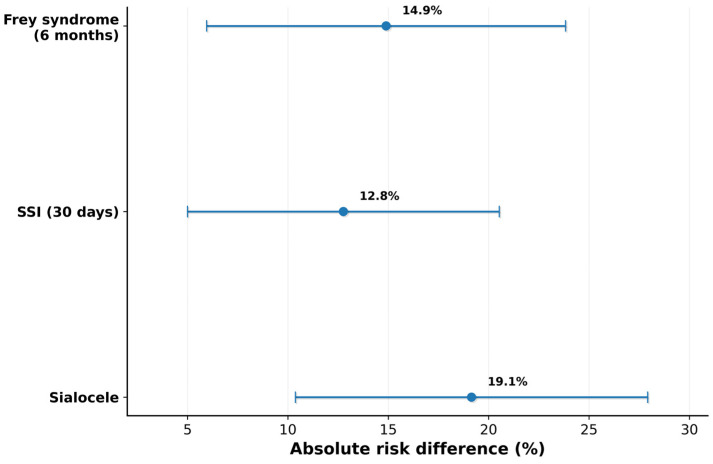
Absolute risk differences for postoperative complications (Group B–Group A). Absolute risk differences (percentage points) with 95% confidence intervals (Newcombe–Wilson) comparing Group B versus Group A for sialocele, surgical-site infection (within 30 days), and Frey syndrome (within 6 months). Negative values indicate lower risk in Group B (combined protocol).

In sensitivity analyses addressing potential unmeasured confounding in this era-defined comparison, E-values for the relative-risk estimates suggested substantial robustness of the association for sialocele (E-value 19.49; upper-CI E-value 4.24), with supportive robustness metrics for surgical-site infection (E-value 13.48; upper-CI E-value 2.66) and Frey syndrome (E-value 8.47; upper-CI E-value 2.54). Absolute risk differences with 95% confidence intervals are displayed in Figure 2.

**Figure 3:**
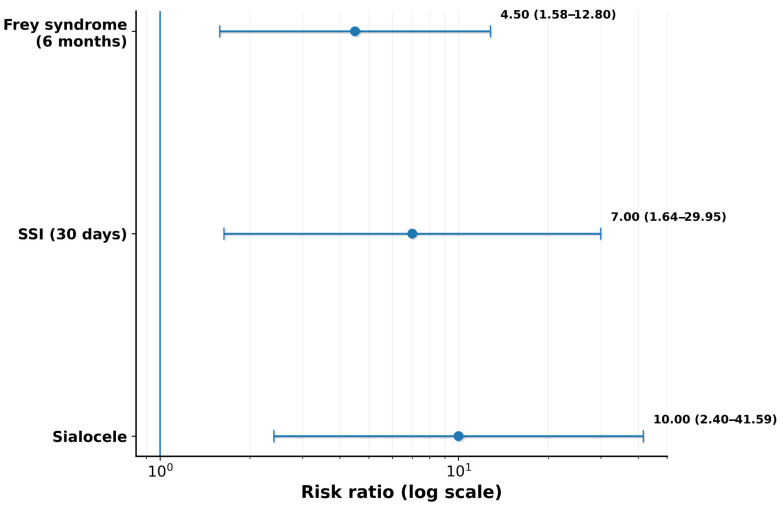
Forest plot of relative risks for primary endpoints. Relative risks (RRs) with 95% confidence intervals (log scale) comparing Group B (combined intraoperative protocol) versus Group A (standard enucleation) for sialocele, surgical-site infection (within 30 days), and Frey syndrome (within 6 months). Values < 1 favor the combined protocol; the vertical blue line indicates RR = 1.

Relative risks with 95% confidence intervals for each endpoint are additionally summarized in a forest plot (Figure 3).

Moreover, the sensitivity to potential unmeasured confounding in this era-defined comparison is summarized using E-values in Table 3.

From a safety standpoint, adjunctive intraoperative BTX-A infiltration and collagen membrane placement did not appear to introduce additional morbidity within the limits of the available dataset. No permanent facial nerve deficits were recorded, and there was no apparent signal suggesting increased perioperative risk attributable to BTX-A infiltration or membrane placement.

## 4. Discussion

In this retrospective comparative cohort of patients undergoing extracapsular enucleation for benign parotid tumors, a standardized intraoperative protocol combining intraparotid botulinum toxin A (BTX-A) infiltration with placement of a bovine collagen membrane over the repaired parotid fascia was associated with a lower postoperative complication burden. Accordingly, these results should be interpreted as hypothesis-generating real-world evidence and warrant prospective validation with protocolized surveillance. The effect was most pronounced for early saliva-related wound morbidity, with a marked reduction in sialocele, and was accompanied by parallel reductions in surgical-site infection and clinically recorded Frey syndrome at 6 months. Although these sequelae are variably reported and may be considered infrequent in some series, they are high-impact complications when they occur: sialocele typically prompts repeated interventions and unplanned care, and Frey syndrome represents a durable late symptom complex with quality-of-life implications. The magnitude of benefit was clinically meaningful, with low numbers needed to treat and concordant effects across endpoints, supporting a pragmatic dual-mechanism strategy: temporary hyposecretion during early wound healing and mechanical separation of tissue planes to mitigate aberrant reinnervation.

The reduction in sialocele is biologically coherent with established BTX-A pharmacology in salivary disorders [26]. By inhibiting acetylcholine release at cholinergic neuroglandular junctions, BTX-A reduces salivary secretion in a localized, reversible manner and has long been used to treat postoperative salivary fistula and sialocele in head and neck practice. Prophylactic intraoperative BTX-A has also been explored as a preventive approach. Different published articles reported fewer salivary complications and lower postoperative drainage without an apparent penalty in facial nerve safety [14,27,28]. Our data extend this concept specifically to conservative enucleation—an increasingly utilized technique that preserves parenchyma and may therefore maintain higher secretory potential in the remnant gland—where early salivary leakage and collections can be particularly consequential for recovery and outpatient burden [29]. This large absolute risk reduction supports the hypothesis that temporary suppression of salivary output during early fascial healing reduces clinically relevant collections requiring aspiration or compression.

The lower SSI rate in the combined protocol group is biologically plausible in the setting of reduced salivary leakage and fluid collections, which may improve wound stability and reduce bacterial proliferation. This interpretation is indirectly supported by the prior literature on salivary complication burden and postoperative morbidity after parotid surgery [11,30,31,32,33]. However, because SSI was not independently adjudicated and allocation was era-based, this finding should be interpreted as supportive rather than causal. With respect to Frey syndrome, our findings align with the prevailing mechanistic framework in which aberrant parasympathetic reinnervation of dermal sweat glands occurs when the parotid bed is not adequately separated from the overlying skin [34,35,36,37,38,39,40,41]. Multiple interpositional strategies, including autologous flaps, fat grafting, and biologic matrices, have been evaluated to reduce Frey syndrome. Systematic reviews and meta-analyses generally support the effectiveness of barrier approaches compared with no interposition, while comparative superiority among techniques is not consistently demonstrated, highlighting that feasibility, reproducibility, operative time, and donor-site morbidity often determine what is most practical in everyday practice [15,42,43,44,45,46,47,48]. Clinical studies of ADM report reductions in subjective symptoms and objective starch–iodine test positivity, with potential benefits on contour deformity [21,49,50,51]. A key limitation is Frey syndrome ascertainment and follow-up. Objective Minor testing was not protocolized and was performed selectively in routine care; therefore, mild or subclinical cases may have been under-detected. In addition, follow-up was limited to 6 months, and Frey syndrome can manifest later. Accordingly, our findings should be interpreted as a reduction in clinically recorded Frey symptoms within 6 months, and prospective studies with scheduled Minor testing and longer follow-up are needed to confirm differences in cumulative incidence. Our data are compatible with this literature and suggest that a collagen-based membrane—applied rapidly and reproducibly without donor-site morbidity—provide a similar “separation” effect. Pairing barrier interposition with temporary salivary suppression may also stabilize the early wound milieu (less leakage, less inflammation, better tissue apposition) and, conceptually, reduce the disorganized regenerative environment that facilitates misdirected nerve growth. This proposed synergy is plausible but remains inferential and should be tested in prospective studies designed to disentangle the independent contributions of BTX-A and membrane interposition. Although the largest absolute effect in this cohort was observed for sialocele, the reduction in Frey syndrome is clinically central because it reflects a late functional sequela that may persist and meaningfully affect long-term postoperative quality of life.

Reported rates of salivary complications and Frey syndrome vary widely across series due to differences in operative extent, use of interposition/barrier techniques, and—critically—endpoint definitions and ascertainment (symptom-based reporting versus protocolized objective screening) [52,53,54,55]. In a parenchyma-sparing setting such as extracapsular enucleation, preserved secretory tissue may increase susceptibility to early salivary collections compared with more extensive resections. Together with symptom-based Frey ascertainment and institution-specific thresholds for documentation, these factors may contribute to higher observed control-group rates relative to studies using different definitions or systematic Minor testing.

A key interpretive consideration is that the protocol was adopted by era (2010–2017 vs. 2018–2025) rather than randomized contemporaneous allocation, making the comparison vulnerable to secular changes in perioperative care, documentation, and surgical practice. We therefore framed the results as associations and reported E-values on the relative-risk scale (Table 3) as a transparent sensitivity analysis for unmeasured confounding. These approaches cannot eliminate confounding, but they improve transparency and help contextualize inference in an era-defined retrospective comparison.

Outcome ascertainment also warrants emphasis, particularly for Frey syndrome. Objective testing with a standardized Minor starch–iodine protocol is often expected in head and neck literature, and selective symptom-driven testing may under-detect mild or subclinical cases. Importantly, the primary endpoints of this study were clinically diagnosed outcomes managed in routine practice and did not require imaging-based surveillance. Although subjective symptoms remain clinically meaningful and guide treatment—especially as botulinum toxin is widely used for symptomatic Frey syndrome [17,18,22]. the absence of protocolized objective screening may bias absolute rates downward and may differ by era if clinical awareness or documentation evolved. Moreover, follow-up was limited to 6 months, and Frey syndrome can emerge later; longer follow-up would better estimate cumulative incidence [14,16,56,57].

Future prospective studies should incorporate scheduled objective Frey testing and longer follow-up to capture cumulative incidence more reliably. Despite these constraints, the present study has pragmatic strengths. The cohort was intentionally homogeneous, restricted to benign pleomorphic adenoma and Warthin tumors treated with a consistent conservative approach, limiting heterogeneity related to tumor biology and extent of resection. The intervention is operationally simple and reproducible, performed at the end of the case without additional incisions or donor-site morbidity, and its clinical impact is readily interpretable through absolute risk reductions and NNT.

Future studies should use prospective contemporaneous controls, protocolized drain metrics and aspiration recording, standardized SSI adjudication, and structured Frey assessment with predefined symptom instruments and scheduled Minor testing. If feasible, factorial or multi-arm designs could clarify the independent contributions of BTX-A and membrane interposition. Systematic perioperative and follow-up data capture may also support transparent AI-enabled prediction models [58,59,60,61,62] for selective use of prophylactic bundles, provided rigorous methodology and standardized outcome ascertainment are maintained.

## 5. Conclusions

In patients undergoing extracapsular enucleation for benign parotid tumors, an intraoperative adjunctive protocol combining intraparotid botulinum toxin A infiltration with bovine collagen membrane interposition over the repaired parotid fascia was associated with a clinically meaningful reduction in postoperative morbidity. The largest benefit was observed for sialocele, with parallel reductions in surgical-site infection and Frey syndrome at 6 months, and no signal of added safety concerns within the limits of this cohort. Given the non-randomized, era-based allocation and the absence of protocolized objective Frey testing, these findings should be interpreted as associative and warrant prospective confirmation. Future studies with contemporaneous controls, standardized outcome ascertainment (including protocolized Minor testing), and detailed perioperative metrics are needed to define the independent and potentially synergistic contributions of temporary salivary suppression and barrier interposition in conservative parotid surgery.

## Figures and Tables

**Table 1:** Patient demographics and tumor characteristics by study cohort.

	Group A (N = 94)	Group B (N = 94)	*p*-Value
Mean age (years)	51.3 ± 11.2	52.1 ± 10.8	0.619
Sex (male/female)	44/50	46/48	0.884
Pleomorphic adenoma	62 (66%)	58 (62%)	0.649
Warthin tumor	32 (34%)	36 (38%)	0.649

Data are presented as mean ± standard deviation for continuous variables and n (%) for categorical variables. Group A: standard extracapsular enucleation (2010–2017). Group B: extracapsular enucleation plus intraoperative intraparotid botulinum toxin A infiltration and bovine collagen membrane placement (2018–2025). *p*-values reflect between-group comparisons (Student’s *t*-test for age; Fisher’s exact test for categorical variables).

**Table 2:** Postoperative outcomes at 6 months with effect size estimates.

	Group A (N = 94)	Group B (N = 94)	Absolute Risk Difference (B−A), % (95% CI)	Relative Risk (95% CI)	Odds Ratio (95% CI)	Fisher *p*
Sialocele	20 (21.3%)	2 (2.1%)	−19.1 (−30.0 to −6.8)	0.100 (0.024–0.416)	0.080 (0.018–0.355)	0.0000478
Surgical-site infection (30 days)	14 (14.9%)	2 (2.1%)	−12.8 (−22.9 to −1.7)	0.143 (0.033–0.611)	0.124 (0.027–0.563)	0.00283
Frey syndrome (6 months)	18 (19.1%)	4 (4.3%)	−14.9 (−26.6 to −2.0)	0.222 (0.078–0.632)	0.188 (0.061–0.578)	0.00247

Outcomes are reported as *n* (%) at 6 months. *p*-values for categorical comparisons are from Fisher’s exact test due to sparse event counts. Absolute risk difference is calculated as Group B–Group A and is expressed as percentage points with 95% confidence intervals (Newcombe–Wilson). Relative risks and odds ratios are provided with 95% confidence intervals.

**Table 3:** Sensitivity to unmeasured confounding (E-values).

Outcome	RR (95% CI)	E-Value (Point Estimate)	E-Value (Upper CI)
Sialocele	0.100 (0.024–0.416)	19.49	4.24
Surgical-site infection (30 days)	0.143 (0.033–0.611)	13.48	2.66
Frey syndrome (6 months)	0.222 (0.078–0.632)	8.47	2.54

E-values quantify the minimum strength of association (on the RR scale) that an unmeasured confounder would need to have with both exposure and outcome to fully explain away the observed association, conditional on measured covariates. For protective associations (RR < 1), E-values are computed using 1/RR. The ‘upper CI’ E-value uses the CI limit closest to the null. E-value formula: E = RR + √{RR(RR − 1)} for RR ≥ 1; for RR < 1, RR was inverted (1/RR) before applying the formula; the ‘upper CI’ E-value uses the CI limit closest to 1.

## Data Availability

The data generated and analyzed during this study are not publicly available due to institutional and privacy policies but are available from the corresponding author upon reasonable request.
